# Supplementary material for: Exploring plant-derived phytochrome chaperone proteins for light-switchable transcriptional regulation in mammals
Source: Nat Commun. 2024 Jun 8;15:4894. doi: 10.1038/s41467-024-49254-5 (PMC11161646; doi:10.1038/s41467-024-49254-5)
Supplement: Supplementary file 1 — Supplementary Information [file 41467_2024_49254_MOESM1_ESM.pdf]

Supplementary Information

Exploring plant-derived phytochrome chaperone proteins for light-switchable transcriptional regulation in mammals

Deqiang Kong[1,4], Yang Zhou[1,2,4], Yu Wei[1,4], Xinyi Wang[1], Qin Huang[1], Xianyun Gao[1], Hang Wan[1], Mengyao Liu[1], Liping Kang[1], Guiling Yu[1], Jianli Yin[1,3], Ningzi Guan[1*], Haifeng Ye[1,2*]

[1]Shanghai Frontiers Science Center of Genome Editing and Cell Therapy, Biomedical Synthetic Biology Research Center, Shanghai Key Laboratory of Regulatory Biology, Institute of Biomedical Sciences and School of Life Sciences, East China Normal University, Dongchuan Road 500, Shanghai 200241, China

[2]Wuhu Hospital, Health Science Center, East China Normal University, Middle Jiuhua Road 263, Wuhu City, China

[3]Chongqing Key Laboratory of Precision Optics, Chongqing Institute of East China Normal University, Chongqing 401120, China

[4]These authors contributed equally.

*Corresponding author: E-mail: hfye@bio.ecnu.edu.cn (H. Y.); nzguan@bio.ecnu.edu.cn (N. G.)

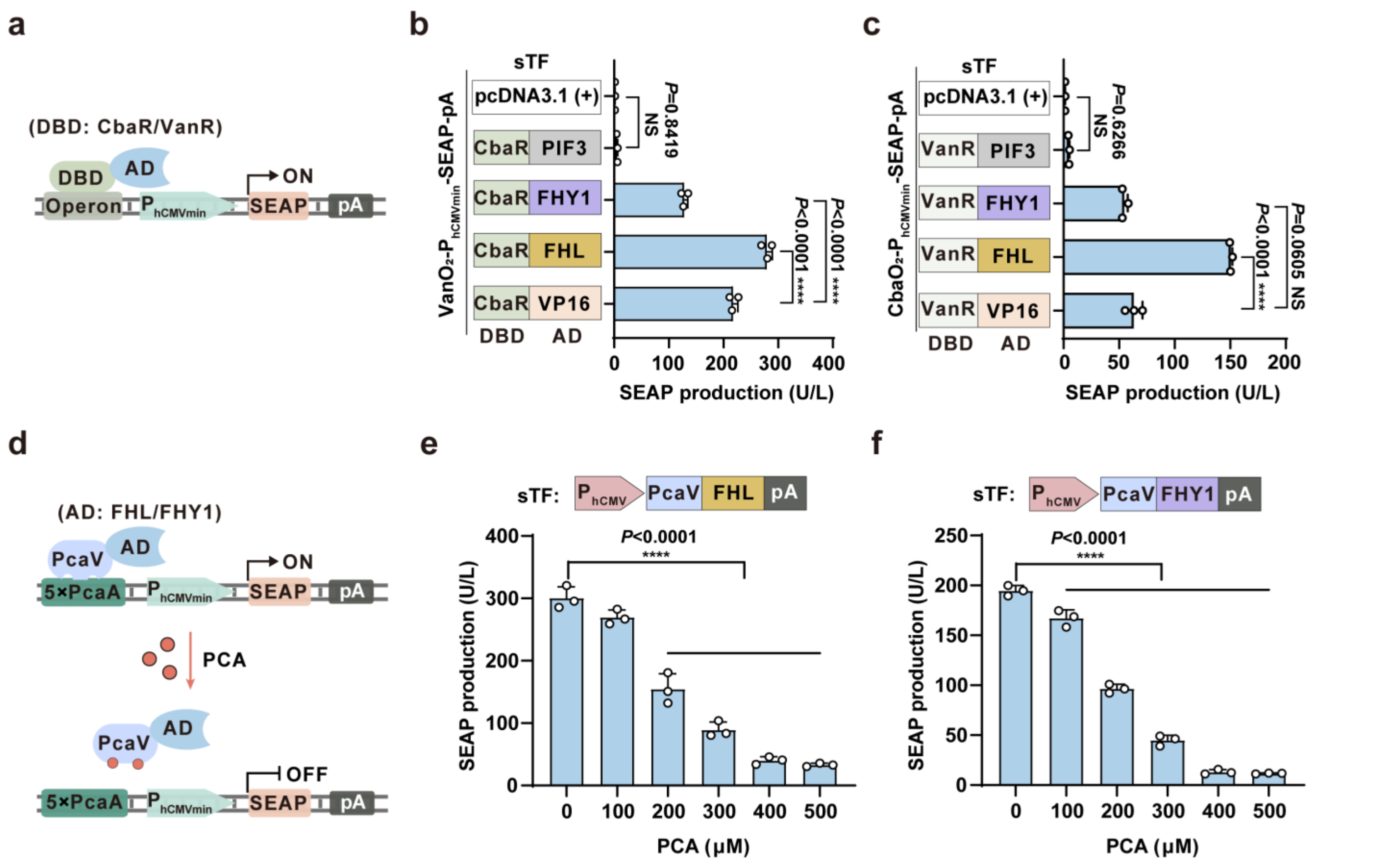

Supplementary Fig. 1 Validation of transcriptional activation functions of the *Arabidopsis*-derived phytochrome chaperone proteins in mammalian cells. a Schematic of the design for screening potential transactivators. The DBD (CbaR or VanR) is combined with the candidate transactivator (PIF3, FHY1, FHL, or VP16) to generate a synthetic TF (sTF), which translocates into the nucleus and binds to the synthetic specific promoter to initiate the target gene expression. **b**, **c** Quantification of gene expression mediated by CbaR-based (**b**) and VanR-based (**c**) sTFs. HEK293T cells (6×10^4) transfected with an sTF alongside the corresponding reporter ($CbaO_2$-$P_{hCMVmin}$-SEAP-pA or $VanO_2$-$P_{hCMVmin}$-SEAP-pA) were grown for 24 hours before quantifying SEAP production. Control cells were transfected with the reporter and pcDNA3.1(+). **d** Schematic of a protocatechuic acid (PCA)-triggered transcriptional inhibition system using FHL/FHY1. Without PCA, the sTF (PcaV-FHL/FHY1) binds to a chimeric $P_{hCMVmin}$ promoter, with a specific DBD binding site positioned upstream of the promoter to initiate target gene expression. In the presence of PCA, PcaV-FHL/FHY1 is released from the chimeric promoter, terminating target gene expression. **e**, **f** Dose-dependent gene expression dynamics facilitated by a PCA-responsive sTF. HEK293T cells (6×10^4) transfected with PcaV-FHL (**e**) or PcaV-FHY1 (**f**), along with the SEAP reporter (O_{PcaA}-$P_{hCMVmin}$-SEAP-pA), were

grown using varying amounts of PCA for 24 hours, before measuring SEAP production. Data are presented as means ± s.d.; $n = 3$ independent experiments, each with 3 technical replicates. Statistical comparisons were performed by one-way ANOVA; NS, not significant, $****p < 0.0001$.

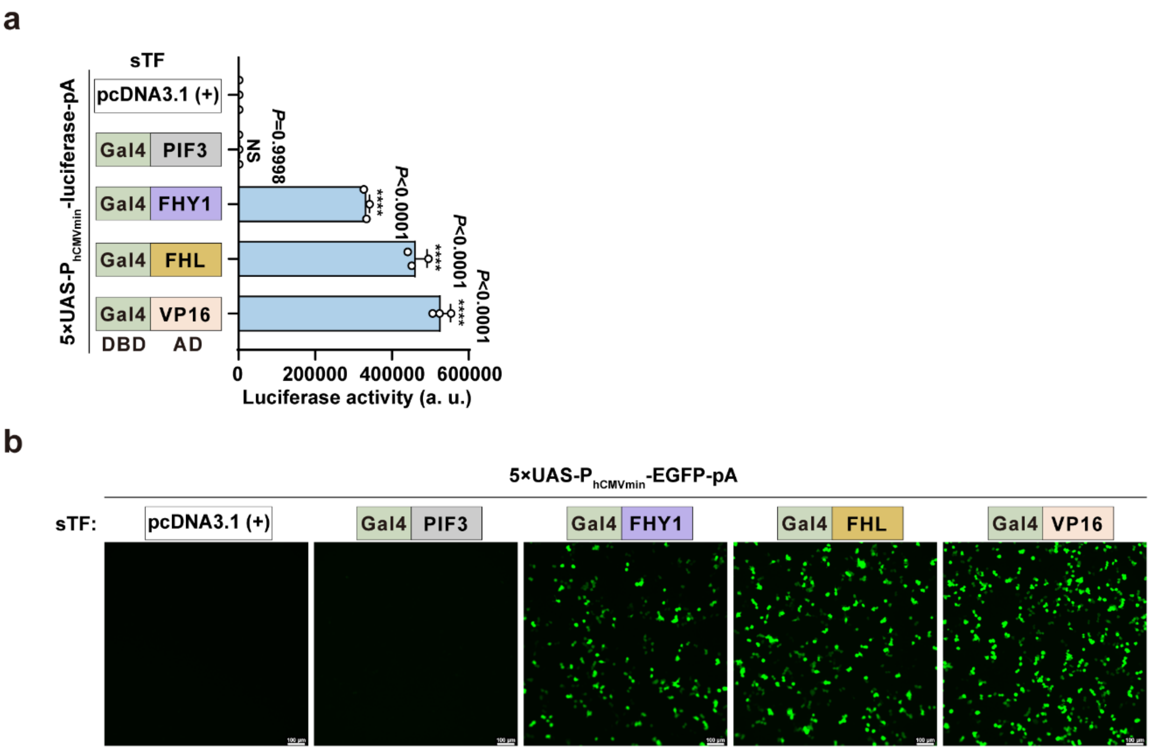

Supplementary Fig. 2 Expression of genes of interest facilitated by different sTFs. a Quantification of luciferase expression mediated by various sTFs. HEK293T cells (6×10^4) transfected with an sTF and the corresponding reporter (5×UAS-$P_{hCMVmin}$-luciferase-pA) were grown for 24 hours before quantifying the luciferase production. Data are presented as means ± s.d.; $n = 3$ independent experiments, each with 3 technical replicates. Statistical comparisons were performed by one-way ANOVA; NS, not significant, $***p < 0.001$, $****p < 0.0001$. **b** Representative fluorescence micrographs of HEK293T cells transfected with different sTFs. HEK293T cells transfected with an sTF and the corresponding reporter (5×UAS-$P_{hCMVmin}$-EGFP-pA) were grown for 24 hours before obtaining fluorescence micrographs. Data are representative of three independent experiments. Scale bar, 100 μm. Control cells were transfected with the reporter and pcDNA3.1(+).

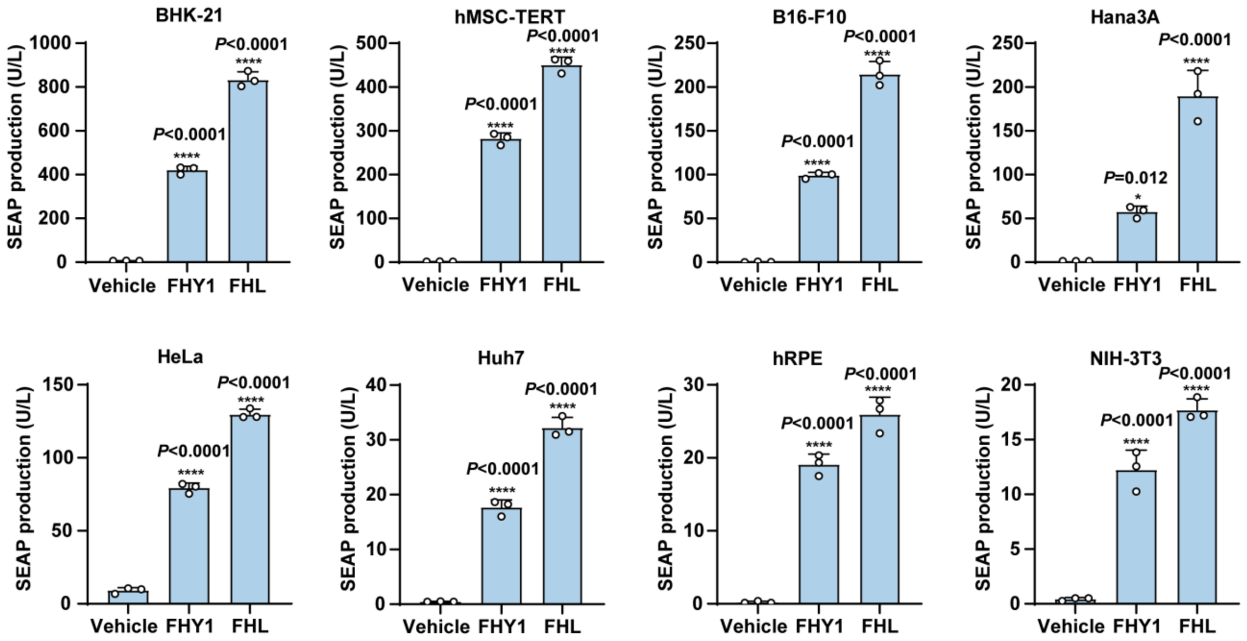

Supplementary Fig. 3 FHL/FHY1-mediated gene expression in diverse mammalian cell lines. Mammalian cells including BHK-21, hMSC-TERT, B16-F10, Hana3A, HeLa, Huh7, hRPE, and NIH-3T3 were transfected using an sTF (Gal4-FHY1 or Gal4-FHL) as well as the corresponding reporter (5×UAS-$P_{hCMVmin}$-SEAP-pA), and SEAP production was quantified 24 hours following transfection. Control cells were transfected with the reporter and pcDNA3.1(+). Data are presented as means ± s.d.; *n* = 3 independent experiments, each with 3 technical replicates. Statistical comparisons were performed by one-way ANOVA; $*p < 0.05$, $****p < 0.0001$.

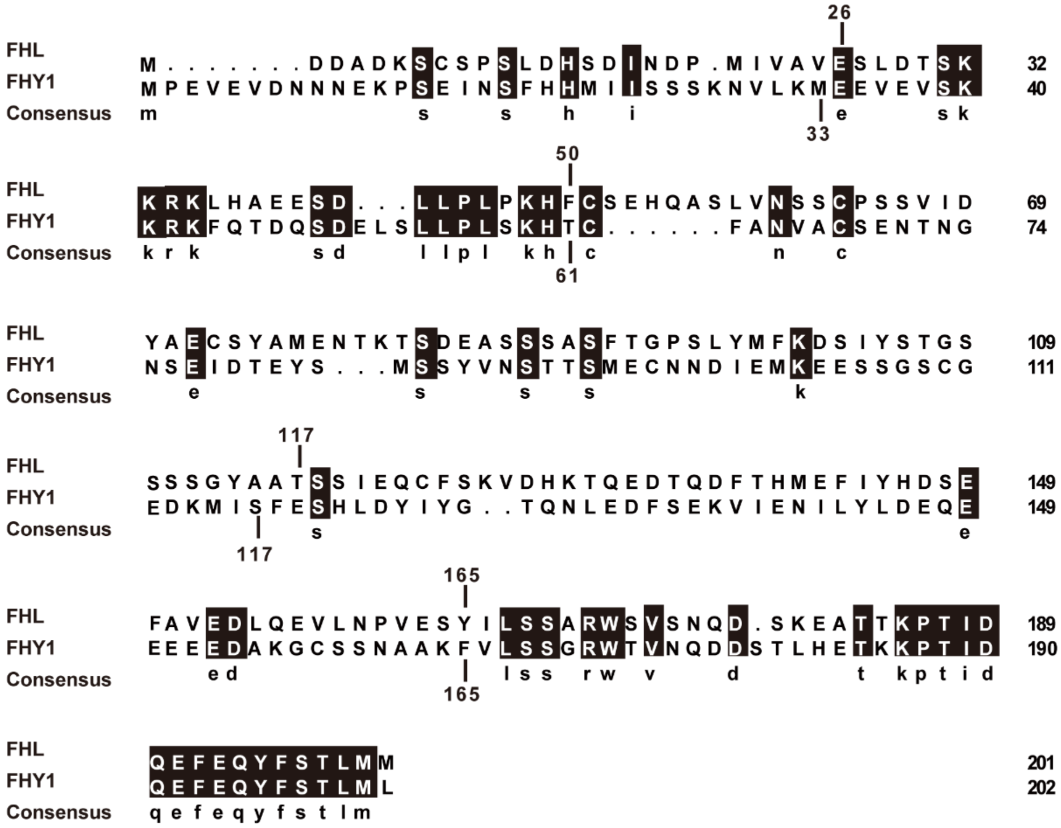

Supplementary Fig. 4 Alignment of amino acid sequences of FHL and FHY1. Identical amino acid residues are indicated in black, and the boundary residue coordinates for each protein are listed at the end. The truncation sites are indicated above (FHL) or below (FHY1) the sequence.

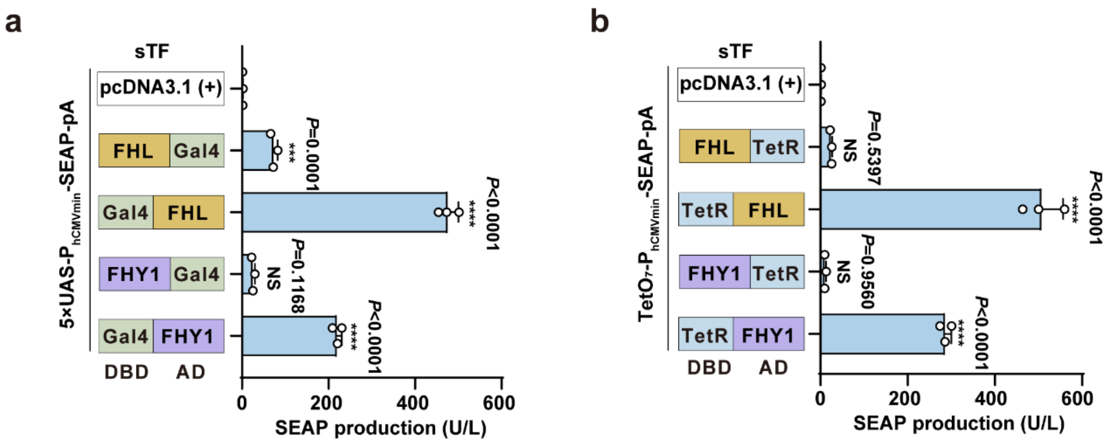

Supplementary Fig. 5 Design and characterization of various sTF constructions for transcriptional activation. sTFs were developed by fusing FHL/FHY1 to the N-terminus or C-terminus of Gal4 (**a**) and TetR (**b**). HEK293T cells (6×10^4) transfected with respective sTFs and the corresponding reporter (5×UAS-$P_{hCMVmin}$-SEAP-pA or $TetO_7$-$P_{hCMVmin}$-SEAP-pA) were grown for 24 hours prior to quantifying SEAP production. Control cells were transfected with the reporter and pcDNA3.1(+). Data are presented as means ± s.d.; n = 3 independent experiments, each with 3 technical replicates. Statistical comparisons were performed by one-way ANOVA; NS, not significant, $***p < 0.001$, $****p < 0.0001$.

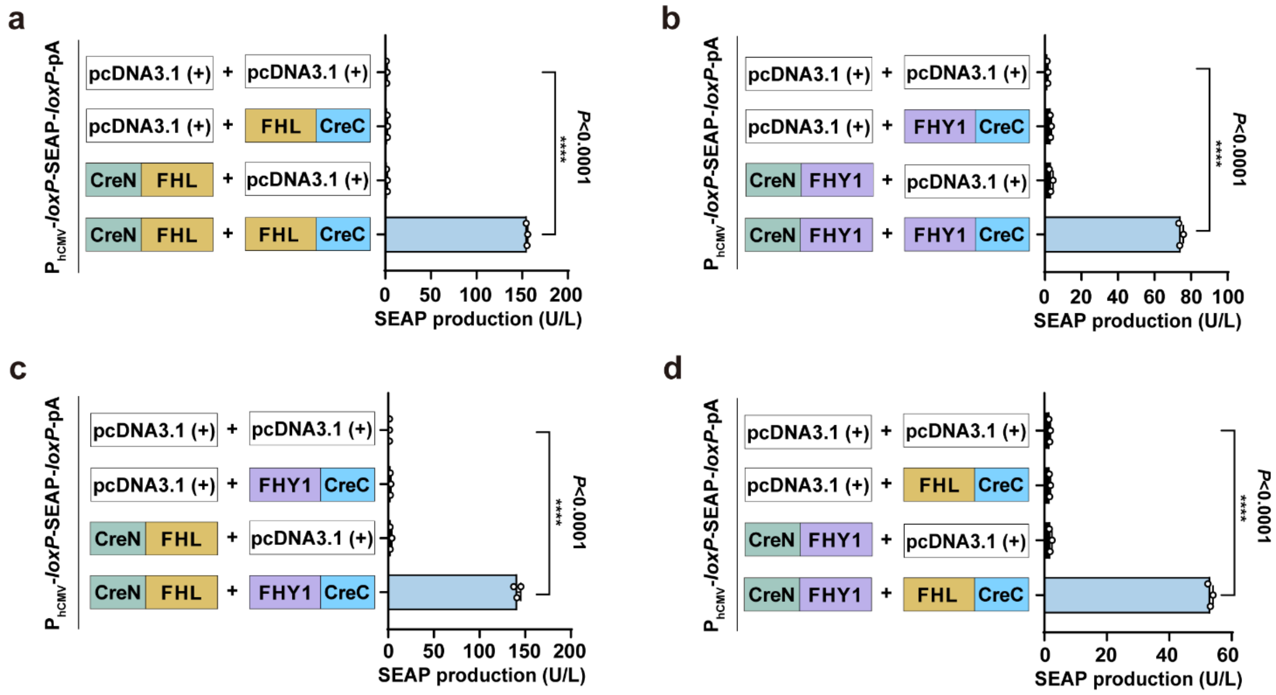

Supplementary Fig. 6 Design and characterization of FHY1/FHL-based split-Cre recombinase systems. a, **b** DNA recombination mediated by homodimerization of full-length FHL (**a**) and FHY1 (**b**). **c**, **d** DNA recombination induced by heterodimerization of full-length FHL and FHY1. HEK293T cells (6×10^4) transfected with CreN/CreC-FHY1/FHL fusions and the corresponding reporter (P_{hCMV}-*loxp*-SEAP-*loxp*-pA) were grown for 24 hours prior to quantifying SEAP production. Control cells were transfected with the reporter and pcDNA3.1(+). Data are presented as means ± s.d.; $n = 3$ independent experiments, each with 3 technical replicates. Statistical comparisons were performed by two-tailed Student's *t*-test; $****p < 0.0001$.

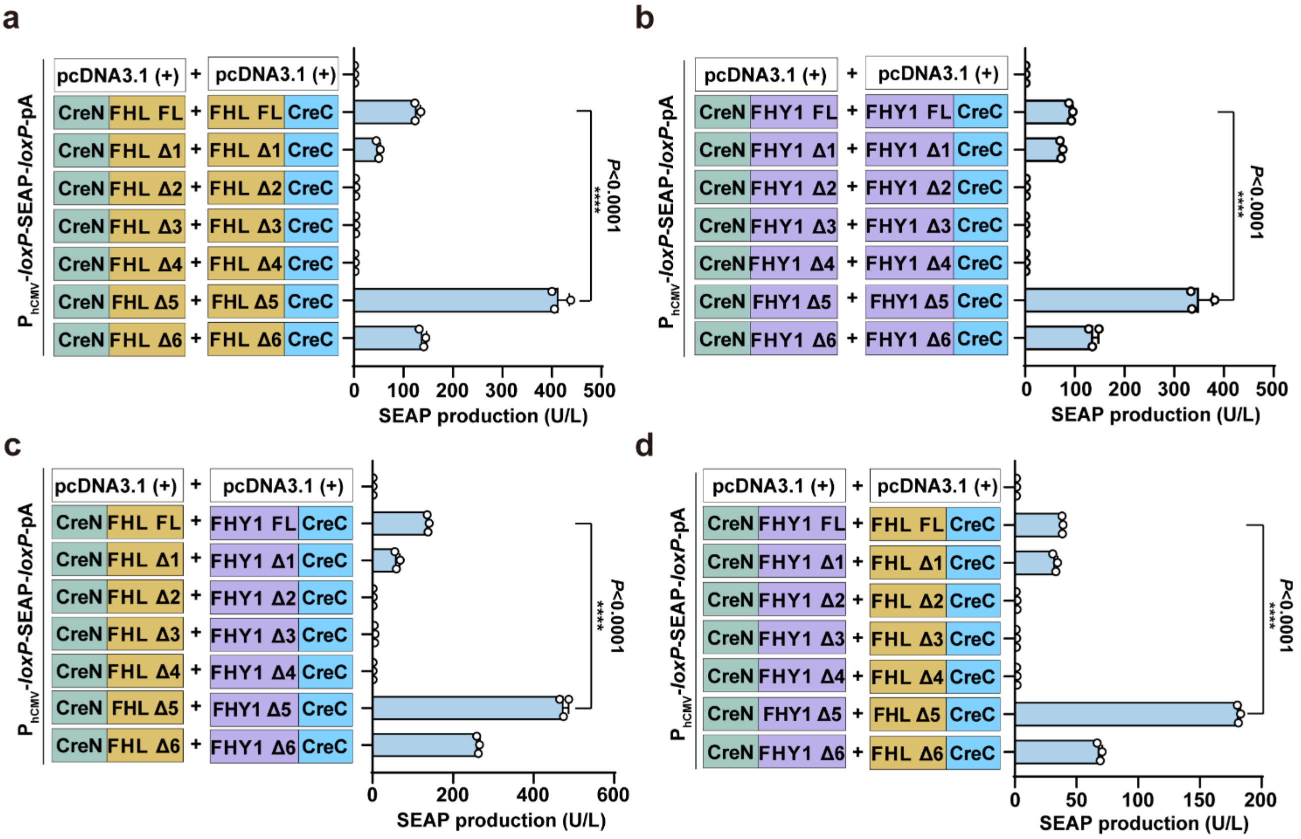

Supplementary Fig. 7 Confirmation of the homo- and/or hetero-dimerization activity of FHY1/FHL truncations with the split-Cre recombinase system. Various FHL and FHY1 truncations were combined with different fragments of split-Cre. HEK293T cells (6×10^4) transfected with respective fusions and the corresponding reporter (P_{hCMV}-*loxp*-SEAP-*loxp*-pA) were grown for 24 hours before quantifying SEAP production. Control cells were transfected with the reporter and pcDNA3.1(+). Data are presented as means ± s.d.; $n = 3$ independent experiments, each with 3 technical replicates. Statistical comparisons were performed by two-tailed Student's *t*-test; ****$p < 0.0001$.

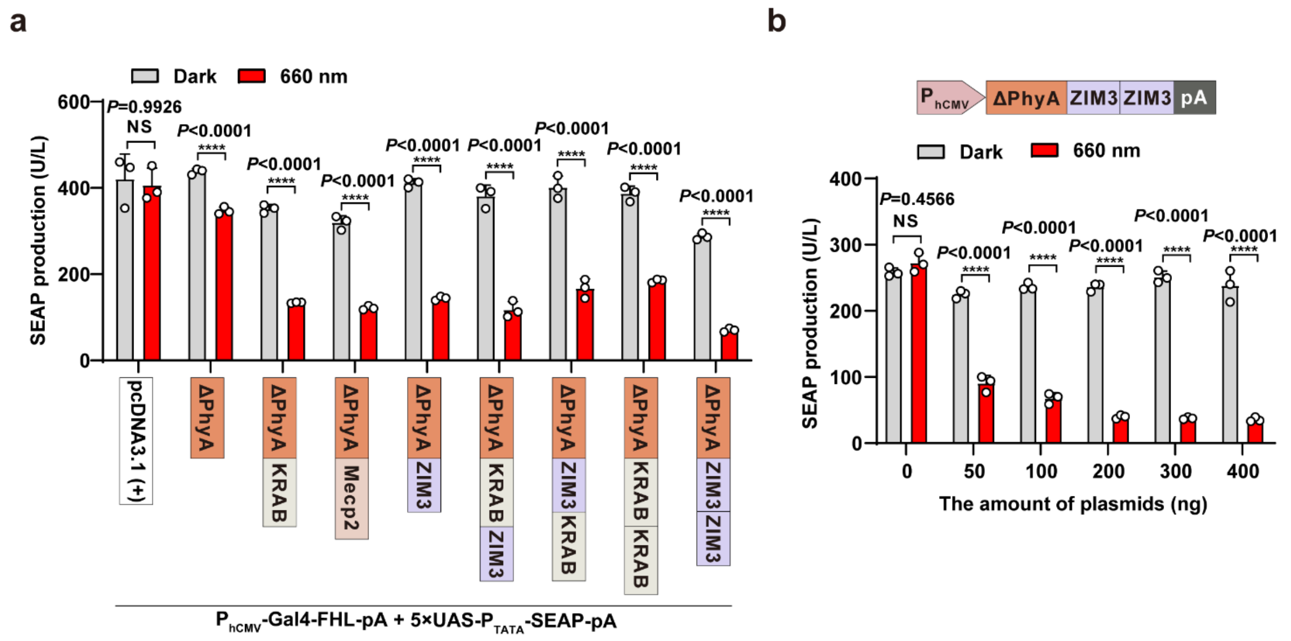

Supplementary Fig. 8 PTRC-mediated transcriptional repression. a Transcriptional repression mediated by a light-responsive transcriptional repressor in different configurations. HEK293T cells (6 × 10^4) transfected with Gal4-FHL, ΔPhyA fused to different transcription repressors, and the corresponding reporter (5×UAS-P_{TATA}-SEAP-pA) were illuminated by red light (660 nm, 1 mW/cm^2) or maintained in darkness for 24 hours prior to quantifying SEAP production. **b** Quantification of PTRC-mediated gene expression with varying amounts of plasmids encoding ΔPhyA-2×ZIM3. Transfected HEK293T cells (6 × 10^4) were illuminated using red light (660 nm, 1 mW/cm^2) or maintained in darkness for 24 hours before quantifying SEAP production. Control cells were transfected with plasmids encoding Gal4-FHL, the corresponding reporter, and pcDNA3.1(+). Data are presented as means ± s.d.; n = 3 independent experiments, each with 3 technical replicates. Statistical comparisons were performed by two-way ANOVA; NS, not significant, $****p < 0.0001$.

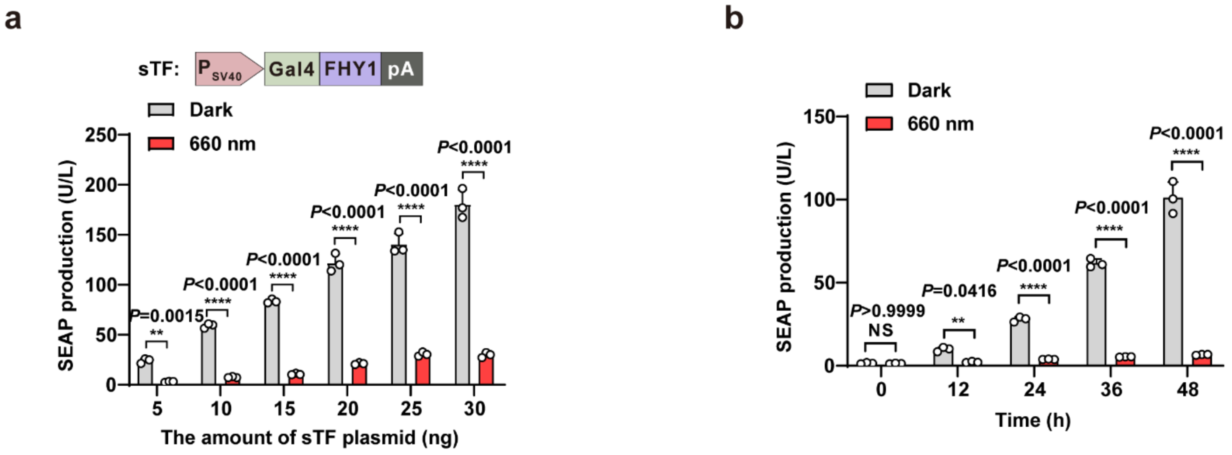

Supplementary Fig. 9 Characterization of PTRC-mediated transcriptional regulation system founded on FHY1. a Quantification of PTRC-mediated gene expression using varying levels of plasmids encoding GAL4-FHY1. Transfected HEK293T cells (6×10^4) were illuminated with red light (660 nm, 1 mW/cm^2) or maintained in darkness for 24 hours before quantifying SEAP production. **b** Quantification of PTRC-mediated gene expression dynamics. Transfected HEK293T cells (6×10^4) were illuminated using red light (660 nm, 1 mW/cm^2) for different time periods as outlined before quantifying SEAP production. Data are presented as means ± s.d.; $n = 3$ independent experiments, each with 3 technical replicates. Statistical comparisons were performed by two-way ANOVA; NS, not significant, $**p < 0.01$, $****p < 0.0001$.

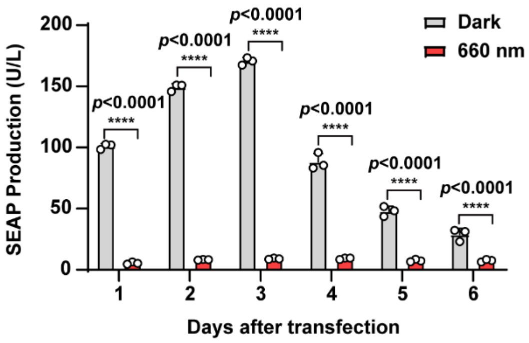

Supplementary Fig. 10 Long-term dynamics of light-responsive transcriptional regulation by the PTRC system. The PTRC transcriptional regulation system was introduced into HEK293T cells using INVI DNA Transfection Reagent. After transfection, the cells were either kept in darkness or exposed to red light (660 nm, 1 mW/cm^2). SEAP production was quantified daily. Data are presented as means ± standard deviation (SD); n = 3 independent experiments, each with 3 technical replicates. Statistical comparisons were performed by two-way ANOVA; ****p < 0.0001.

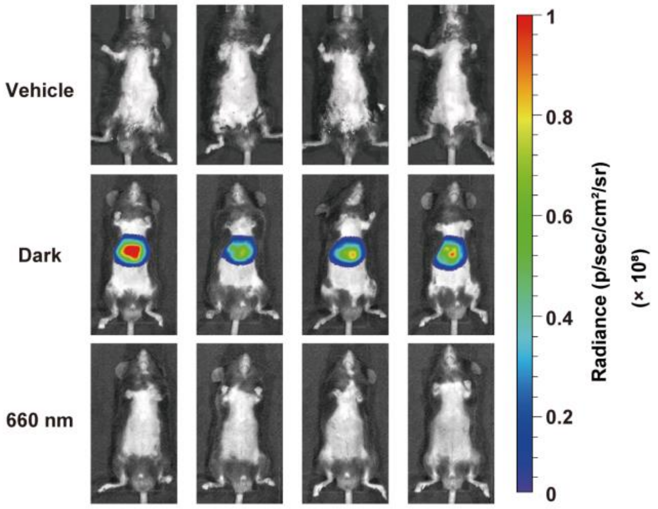

Supplementary Fig. 11 Detailed images of bioluminescence in mice possessing a PTRC-mediated transcriptional regulation system. Plasmids encoding ΔPhyA-2×ZIM3, Gal4-FHL, and the corresponding luciferase reporter were delivered into the mouse liver by hydrodynamic injection of the tail vein. Control mice were administered pcDNA3.1(+) instead of Gal4-FHL. The mice were intraperitoneally injected with PCB and exposed to red light (660 nm, 5 mW/cm^2, 1 minute on, 5 minutes off, alternating) or continuous darkness for 16 hours before the bioluminescence images of mice were captured using an IVIS.

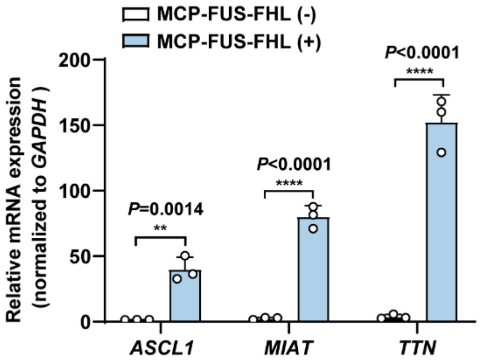

Supplementary Fig. 12 MCP-FUS-FHL-mediated endogenous gene activation. HEK293T cells (6 × 10^4) were transfected with plasmids encoding dCas9, MCP-FUS-FHL, and sgRNA targeting different endogenous genes (*ASCL1*, *MIAT*, or *TTN*). The relative mRNA expression was determined using qPCR at 48 hours after transfection. Data are presented as means ± s.d.; n = 3 independent experiments, each with 3 technical replicates. Statistical comparisons were performed by two-way ANOVA; $^{**}p < 0.01$, $^{****}p < 0.0001$.

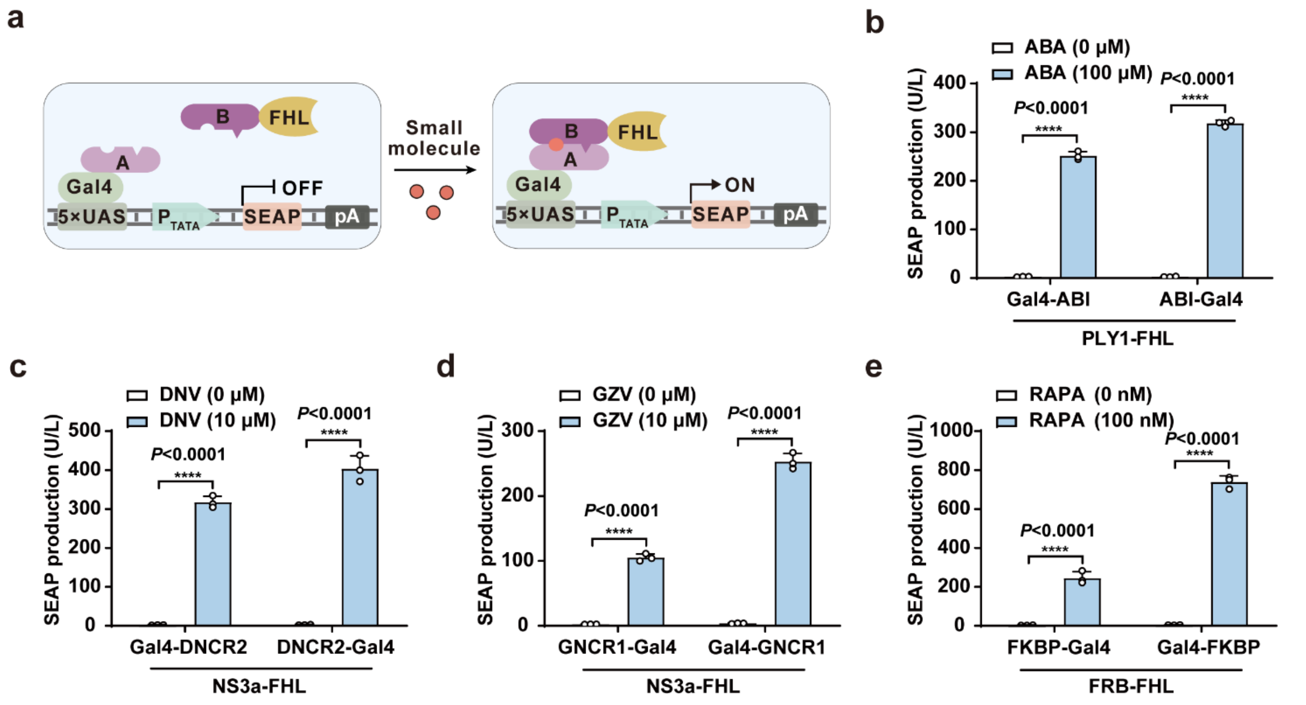

Supplementary Fig. 13 Design and characterization of small molecule-induced transcriptional regulation systems based on FHL. a Schematic of small molecule-induced transcriptional regulation systems founded on FHL. Gal4-based synthetic DBD (ABI-Gal4, DNCR2-Gal4, Gal4-GNCR1, or Gal4-FKBP) binds to the chimeric promoter (5×UAS-P_{TATA}). In the presence of small molecules, including ABA (abscisic acid), DNV (Danoprevzr), GZV (Grazoprevir), or RAPA (Rapamycin), the small molecule-responsive transactivator (PYL1-FHL, NS3a-FHL, or FRB-FHL) is recruited to the corresponding promoter according to interactions with the synthetic DBD to initiate target gene expression. **b-e** Quantification of FHL-mediated transcriptional activation mediated by ABA (abscisic acid) (**b**), DNV (Danoprevzr) (**c**), GZV (Grazoprevir) (**d**), and RAPA (Rapamycin) (**e**). Transfected HEK293T cells (6×10^4) were grown for 24 hours in the presence or absence of small molecule ligands before quantifying SEAP production. Data in (**b**, **c**, **d**, **e**) are presented as means ± s.d.; $n = 3$ independent experiments, each with 3 technical replicates. Statistical comparisons were performed by two-way ANOVA; ****$p < 0.0001$.

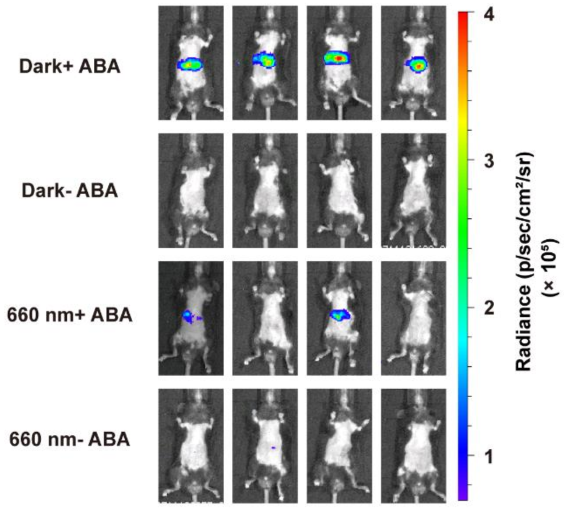

Supplementary Fig. 14 Detailed images of bioluminescence in mice possessing a PTRC$_{CIP\text{-}ABA}$-mediated transcriptional regulation system. Plasmids encoding the PTRC$_{CIP\text{-}ABA}$ system (ABI-Gal4, PYL1-FHL, and ΔPhyA-2×ZIM3) and luciferase reporter (5×UAS-P$_{hCMVmin}$-luciferase-pA) were administered to the mouse liver via hydrodynamic tail vein injection. The mice were intraperitoneally injected with ABA eight hours later and illuminated with red light for 16 hours or maintained in darkness. The bioluminescence signal was measured using an IVIS 24 hours after hydrodynamic injection.

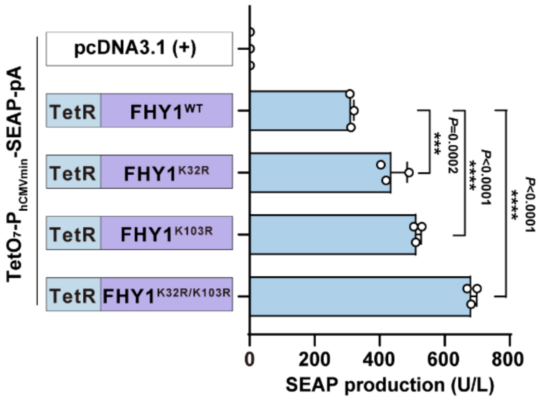

Supplementary Fig. 15 Transcriptional activation of FHY1 variants. Mutations were introduced into various SUMOylation sites of FHY1, resulting in K32R and/or K103R. These FHY1 variants were linked to TetR and transfected into HEK293T cells alongside the corresponding SEAP reporter. After 24 hours of incubation, the production of SEAP was quantified. SUMOylation motifs (LK^{32}ME and MK^{103}EE) were identified using JASSA (http://www.jassa.fr/index.php?m=jassa). Data are presented as means ± s.d.; n = 3 independent experiments, each with 3 technical replicates. Statistical comparisons were performed by one-way ANOVA; ***$p < 0.001$, ****$p < 0.0001$.

Supplementary Table 1. DNA sequence data.

**FHL**
**atgGACGACGCCGACAAGAGCTGCAGCCCCTCTTTAGACCACTCCGACATCAACGATCCCATGATC<br>GTGGCCGTGGAGTCTTTAGACACAAGCAAGAAGAGGAAGCTGCACGCCGAAGAGTCCGATTTACT<br>GCCTTTACCCAAGCACTTCTGCAGCGAGCACCAAGCTTCTTTAGTGAACAGCAGCTGCCCCAGCA<br>GCGTGATCGACTACGCCGAGTGCAGCTACGCCATGGAGAACACCAAAACCAGCGACGAGGCCTCC<br>AGCAGCGCTAGCTTCACCGGACCCTCTTTATACATGTTCAAGGACAGCATCTACAGCACTGGTAG<br>CAGCTCCTCCGGCTACGCTGCCACCAGCAGCATCGAGCAGTGCTTCTCCAAGGTGGACCACAAGA<br>CCCAAGAAGACACCCAAGATTTCACCCACATGGAGTTCATCTACCACGACAGCGAGTTTGCCGTG<br>GAGGATTTACAAGAAGTGCTGAACCCCGTTGAGTCCTACATTTTAAGCAGCGCCAGATGGAGCGT<br>CTCCAACCAAGATAGCAAGGAGGCCACCACCAAACCCACCATCGACCAAGAATTCGAGCAGTACT<br>TCAGCACTTTAATGATGtaa**
**FHY1**
**atgCCCGAGGTGGAGGTCGACAACAACAACGAGAAGCCCAGCGAGATCAACAGCTTCCACCACATG<br>ATTATCAGCAGCAGCAAGAACGTGCTGAAGATGGAGGAGGTGGAGGTGTCCAAGAAGAGGAAGT<br>TCCAGACCGACCAGAGCGACGAGCTGAGCTTATTACCTTTATCCAAACACACATGCTTTGCCAACG<br>TGGCTTGTAGCGAGAACACCAACGGCAACAGCGAGATCGATACCGAGTACAGCATGTCCAGCTAC<br>GTGAACTCCACCACCTCCATGGAGTGCAACAACGACATCGAGATGAAGGAAGAATCCAGCGGCAG<br>CTGCGGAGAGGACAAGATGATCAGCTTCGAGTCCCATTTAGACTATATCTACGGCACCCAGAATC<br>TGGAGGACTTCTCCGAGAAGGTGATCGAGAACATTCTGTACCTCGACGAGCAAGAAGAGGAGGAG<br>GAGGACGCCAAGGGCTGTAGCAGCAACGCTGCCAAGTTCGTGCTGTCCTCTGGTCGTTGGACCGT<br>GAACCAAGATGATAGCACTTTACACGAAACCAAGAAGCCCACCATCGACCAAGAATTCGAGCAGT<br>ACTTTAGCACTTTAATGCTGtaa**
**ΔPhyA**
**atgGAGAAGAAGATGAGCGGATCTCGTCCCACACAGTCCAGCGAGGGATCTCGTAGATCTCGTCAC<br>TCCGCTCGTATCATCGCTCAGACCACCGTGGACGCCAAACTGCACGCCGATTTCGAGGAGAGCGG<br>CTCCTCCTTTGATTACTCCACCAGCGTGAGGGTGACTGGTCCCGTGGTCGAGAACCAGCCTCCTA<br>GGAGCGACAAGGTCACCACAACCTACCTCCATCATATCCAGAAGGGCAAGCTGATCCAGCCCTTT<br>GGATGTTTACTGGCTTTAGACGAAAAGACCTTCAAGGTCATCGCCTACTCCGAGAACGCCTCCGA<br>ACTGCTCACAATGGCTTCCCACGCCGTGCCTAGCGTGGGAGAGCACCCCGTTCTGGGCATCGGCA<br>CCGATATCAGATCTTTATTTACCGCTCCCAGCGCTTCCGCTTTACAGAAGGCTCTCGGCTTCGGCG<br>ACGTGTCTTTACTGAACCCTATCCTCGTCCATTGTCGTACATCCGCCAAGCCCTTCTACGCCATTA<br>TCCATAGGGTGACCGGATCCATCATCATCGACTTCGAACCCGTTAAGCCCTATGAAGTGCCCATG<br>ACAGCCGCCGGAGCTTTACAGAGCTATAAGCTGGCCGCCAAGGCCATCACCAGACTCCAGTCTTT<br>ACCCAGCGGAAGCATGGAGAGGCTGTGCGACACCATGGTGCAAGAAGTCTTCGAGCTGACTGGTT<br>ACGATCGTGTCATGGCCTACAAGTTCCACGAGGACGACCACGGCGAGGTGGTGAGCGAAGTGAC<br>CAAGCCCGGTTTAGAACCCTATTTAGGTTTACACTATCCCGCCACAGACATCCCCCAAGCTGCCAG<br>ATTTTTATTTATGAAGAACAAGGTGAGAATGATCGTGGACTGCAACGCCAAGCACGCTAGGGTTTT<br>ACAAGATGAGAAGCTGAGCTTCGATTTAACCCTCTGCGGCAGCACTTTAAGAGCTCCCCACTCTTG<br>TCATCTGCAATACATGGCCAATATGGACAGCATCGCTTCTTTAGTGATGGCCGTGGTGGTGAATG<br>AGGAGGATGGAGAGGGCGATGCTCCCGATGCCACCACACAGCCTCAGAAGAGAAAGAGGCTGTG<br>GGGTTTAGTGGTCTGCCACAACACCACCCCCAGATTTGTCCCTTTTCCCTTACGTTATGCTTGTGA<br>ATTTTTAGCCCAAGTGTTTGCCATCCACGTCAACAAGGAGGTCGAGCTGGACAACCAGATGGTGG**

**AGAAGAACATTTTACGTACCCAGACACTCCTCTGCGACATGCTCATGAGGGACGCTCCCCTCGGCATCGTGAGCCAGTCCCCCAATATTATGGATTTAGTCAAGTGCGACGGCGCCGCCTTATTATACAAGGACAAGATCTGGAAGCTGGGCACCACCCCTAGCGAGTTCCATTTACAAGAAATCGCTTCTTGGCTGTGTGAGTACCACATGGATTCCACCGGTTTAAGCACCGATTCTTTACACGACGCTGGCTTTCCTAGGGCTTTATCTTTAGGCGACAGCGTCTGCGGAATGGCTGCCGTTCGTATCAGCAGCAAAGATATGATTTTCTGGTTTCGTTCCCATACCGCCGGCGAAGTGAGATGGGGCGGCGCCAAACACGACCCCGATGATAGGGACGATGCCAGAAGGATGCACCCCAGATCCTCCTTCAAGGCTTTTCTGGAGGTGGTGAAGACCAGAAGCTTACCTTGGAAGGACTACGAAATGGATGCCATCCACTCTTTACAGCTCATCTTACGTAACGCCTTTAAGGACAGCGAGACCACCGACGTGAACACAAAGGTGATCTATAGCAAGCTCAACGATCTCAAGATTGATGGAATCCAAtaa**
**ZIM3**
**atgAACAATTCCCAGGGAAGAGTGACCTTCGAGGATGTCACTGTGAACTTCACCCAGGGGGAGTGGCAGCGGCTGAATCCCGAACAGAGAAACTTGTACAGGGATGTGATGCTGGAGAATTACAGCAACCTTGTCTCTGTGGGACAAGGGGAAACCACCAAACCCGATGTGATCTTGAGGTTGGAACAAGGAAAGGAGCCATGGTTGGAGGAAGAGGAAGTGCTGGGAAGTGGCCGTGCAGAAAAAAATGGGGACATTGGAGGGCAGATTTGGAAGCCAAAGGATGTGAAAGAGAGTCTtaa**

Supplementary Table 2. Target sequences of sgRNAs employed in this study.

Gene name	Guide sequence of sgRNA (5'-3')
ASCL1	GGCTGGGTGTCCCATTGAAA
	ATGGAGAGTTTGCAAGGAGC
RHOXF2	ACGCGTGCTCTCCCTCATC
	CTGTGGGTTGGGCCTGCTG
TTN	CCTTGGTGAAGTCTCCTTTG
	ATGTTAAAATCCGAAAATGC
MIAT	GCGCCCATGAAATTTTAATG
	GCTTCTGCGCCCCTGGTCCG
Ascl1 (mouse)	GCAGCCGCTCGCTGCAGCAG
	AGCTGAGGAGGTGGGGGAAG
Hbb (mouse)	AGAGAGTCTGGGCAAGACAG
Ins2 (mouse)	GTTAAGACTCTAATTACCCT
Dkk1 (mouse)	CCCAAAGCTTTGAAATCCCA

Supplementary Table 3. Oligonucleotide sequences used for qPCR analysis.

Gene name	Primer name	Primer sequence (5'-3')
ASCL1 (human)	Forward	CGCGGCCAACAAGAAGATG
	Reverse	CGACGAGTAGGATGAGACCG
GAPDH (human)	Forward	CGAGATCCCTCCAAAATCAA
	Reverse	ATCCACAGTCTTCTGGGTGG
RHOXF2 (human)	Forward	AGTGTAGCCAGTATATGACCAGC
	Reverse	TGACCTCTTCAGTAAGCGACAG
TTN (human)	Forward	CCCCATCGCCCATAAGACAC
	Reverse	CCACGTAGCCCTCTTGCTTC
MIAT (human)	Forward	TGGCTGGGGTTTGAACCTTT
	Reverse	AGGAAGCTGTTCCAGACTGC
Ascl1 (mouse)	Forward	GGAACAAGAGCTGCTGGACT
	Reverse	GTTTTTCTGCCTCCCCATTT
Gapdh (mouse)	Forward	ATGACATCAAGAAGGTGGTG
	Reverse	CATACCAGGAAATGAGCTTG
Hbb (mouse)	Forward	CACTTGAGATCATCTCCAAGC
	Reverse	TAACCCCCAAGCCCAAGGATG
Ins2 (mouse)	Forward	GGCTTCTTCTACACACCCAT
	Reverse	CCAAGGTCTGAAGGTCACCT
Dkk1 (mouse)	Forward	TCCGTCTGCCTCCGATCATC
	Reverse	GCCTTTCCGTTTGTGCTTGG
tdTomato (mouse)	Forward	GACACCAAGCTGGACATCAC
	Reverse	ACCTTGAAGCGCATGAACTC
