# Supplementary material for: Exploring plant-derived phytochrome chaperone proteins for light-switchable transcriptional regulation in mammals
Source: Nat Commun. 2024 Jun 8;15:4894. doi: 10.1038/s41467-024-49254-5 (PMC11161646; doi:10.1038/s41467-024-49254-5)
Supplement: Supplementary file 2 — Reporting Summary [file 41467_2024_49254_MOESM2_ESM.pdf]

nature portfolio

Corresponding author(s): Haifeng Ye

Last updated by author(s): May 25, 2024

Reporting Summary

Nature Portfolio wishes to improve the reproducibility of the work that we publish. This form provides structure for consistency and transparency in reporting. For further information on Nature Portfolio policies, see our Editorial Policies and the Editorial Policy Checklist.

Statistics

For all statistical analyses, confirm that the following items are present in the figure legend, table legend, main text, or Methods section.

n/a	Confirmed	
☐	☒	The exact sample size (*n*) for each experimental group/condition, given as a discrete number and unit of measurement
☐	☒	A statement on whether measurements were taken from distinct samples or whether the same sample was measured repeatedly
☐	☒	The statistical test(s) used AND whether they are one- or two-sided<br>*Only common tests should be described solely by name; describe more complex techniques in the Methods section.*
☒	☐	A description of all covariates tested
☒	☐	A description of any assumptions or corrections, such as tests of normality and adjustment for multiple comparisons
☐	☒	A full description of the statistical parameters including central tendency (e.g. means) or other basic estimates (e.g. regression coefficient) AND variation (e.g. standard deviation) or associated estimates of uncertainty (e.g. confidence intervals)
☐	☒	For null hypothesis testing, the test statistic (e.g. *F*, *t*, *r*) with confidence intervals, effect sizes, degrees of freedom and *P* value noted<br>*Give P values as exact values whenever suitable.*
☒	☐	For Bayesian analysis, information on the choice of priors and Markov chain Monte Carlo settings
☒	☐	For hierarchical and complex designs, identification of the appropriate level for tests and full reporting of outcomes
☒	☐	Estimates of effect sizes (e.g. Cohen's *d*, Pearson's *r*), indicating how they were calculated

Our web collection on statistics for biologists contains articles on many of the points above.

Software and code

Policy information about availability of computer code

Data collection	SEAP and Luciferase expression level in culture media were quantified using a Synergy H1 hybrid multi-mode microplate reader with Gen5 software (version: 2.04).<br>qPCR reaction were performed on the LightCycler 96 real-time PCR instrument (Roche, Switzerland).<br>Western Blot images were visualized using a fluorescent western blot imaging system (LI-COR Odyssey Biosciences, USA).<br>Fluorescence image of EGFP and tdTomato expression cells were performed with an inverted fluorescence microscope (Olympus IX71, TH4-200, Olympus, Japan).<br>Bioluminescence images of the mice were obtained using IVIS Lumina II in vivo imaging system (Perkin Elmer, USA).
Data analysis	We used GraphPad (version 8) to perform statistical analysis.<br>We used Gen5 software (version 2.04) to analyze absorbances of different samples.<br>Fluorescence intensity of EGFP expression cells was analyzed using ImageJ® 1.51a software.<br>Bioluminescence values of the mice were analyzed using Living Image® software (version 4.3.1) .

For manuscripts utilizing custom algorithms or software that are central to the research but not yet described in published literature, software must be made available to editors and reviewers. We strongly encourage code deposition in a community repository (e.g. GitHub). See the Nature Portfolio guidelines for submitting code & software for further information.

Data

Policy information about availability of data

All manuscripts must include a data availability statement. This statement should provide the following information, where applicable:

- Accession codes, unique identifiers, or web links for publicly available datasets
- A description of any restrictions on data availability
- For clinical datasets or third party data, please ensure that the statement adheres to our policy

All data associated with this study are present in the paper or the Supplementary Information. All genetic components related to this paper are available with a material transfer agreement and can be requested from H.Y. (hfye@bio.ecnu.edu.cn). Source data are provided with this paper.

Research involving human participants, their data, or biological material

Policy information about studies with human participants or human data. See also policy information about sex, gender (identity/presentation), and sexual orientation and race, ethnicity and racism.

Reporting on sex and gender	No human subjects were used.
Reporting on race, ethnicity, or other socially relevant groupings	None.
Population characteristics	None.
Recruitment	None.
Ethics oversight	None.

Note that full information on the approval of the study protocol must also be provided in the manuscript.

Field-specific reporting

Please select the one below that is the best fit for your research. If you are not sure, read the appropriate sections before making your selection.

[x] Life sciences [ ] Behavioural & social sciences [ ] Ecological, evolutionary & environmental sciences

For a reference copy of the document with all sections, see nature.com/documents/nr-reporting-summary-flat.pdf

Life sciences study design

All studies must disclose on these points even when the disclosure is negative.

Sample size	No statistical methods were used to predetermine sample size. For vitro study, at least three biologically independent experimental replicates were performed for statistical analyses, according to standard scientific conventions. For in vivo study, n=4 or n=5 or n=6 mouse per group were used are based on the need for proper statistical evaluation.
Data exclusions	No data were excluded.
Replication	The number of independent experiments is specified in each figure legend, with at least 3 independent experiments, unless otherwise specified.
Randomization	All the animals and cells were randomly assigned to experimental groups.
Blinding	For all the animal and cell experiments, the investigators were blinded to the group allocations during data analysis.

Reporting for specific materials, systems and methods

We require information from authors about some types of materials, experimental systems and methods used in many studies. Here, indicate whether each material, system or method listed is relevant to your study. If you are not sure if a list item applies to your research, read the appropriate section before selecting a response.

Materials & experimental systems

n/a	Involved in the study
☐	☒ Antibodies
☐	☒ Eukaryotic cell lines
☒	☐ Palaeontology and archaeology
☐	☒ Animals and other organisms
☒	☐ Clinical data
☒	☐ Dual use research of concern
☒	☐ Plants

Methods

n/a	Involved in the study
☒	☐ ChIP-seq
☒	☐ Flow cytometry
☒	☐ MRI-based neuroimaging

Antibodies

Antibodies used	anti-tdTomato primary antibody (A00682, GenScript)<br>anti-GAPDH primary antibody (AF1186, Beyotime)
Validation	All antibodies were validated for the specified application by respective manufacturer.

Eukaryotic cell lines

Policy information about cell lines and Sex and Gender in Research

Cell line source(s)	Hana3A cells and human mesenchymal stem cells (hMSC-TERT) were obtained from Professor Dr. Martin Fussenegger.<br>The HEK293T, BHK-21, NIH-3T3 cells were obtained from ATCC.<br>The HeLa, Huh7, ARPE-19 cells were obtained from Chinese Academy of Sciences.
Authentication	None of the cell lines are authenticated.
Mycoplasma contamination	No mycoplasma contamination was detected for all cell lines.
Commonly misidentified lines<br>(See ICLAC register)	No misidentified cell lines have been used in this study.

Animals and other research organisms

Policy information about studies involving animals; ARRIVE guidelines recommended for reporting animal research, and Sex and Gender in Research

Laboratory animals	C57BL/6J mice (male, 8-week-old) were purchased from the ECNU (East China Normal University) Laboratory Animal Center. Gt (ROSA)26Sortm14(CAG-tdTomato)Hze mice were a gift from Professor Dr. Dali Li. All the mice were kept on a standard alternating 12-h light/12-h dark cycle and were given a normal chow diet (6% fat and 18% protein (wt/wt)) and water .
Wild animals	None.
Reporting on sex	All studies using C57BL/6J and Gt(ROSA)26Sortm14(CAG-tdTomato)Hze mice employed male mice.
Field-collected samples	None.
Ethics oversight	The protocol involved in this study was approved by the ECNU Animal Care and Use Committee (protocol ID: m20220412, m20220506)

Note that full information on the approval of the study protocol must also be provided in the manuscript.

Plants

Seed stocks	None.
Novel plant genotypes	None.
Authentication	None.
