# Supplementary material for: Exploring plant-derived phytochrome chaperone proteins for light-switchable transcriptional regulation in mammals
Source: Nat Commun. 2024 Jun 8;15:4894. doi: 10.1038/s41467-024-49254-5 (PMC11161646; doi:10.1038/s41467-024-49254-5)
Supplement: Supplementary file 6 — Description of Additional Supplementary Files [file 41467_2024_49254_MOESM6_ESM.pdf]

Description of Additional Supplementary Files

File Name: Supplementary Data 1
Description: Plasmids designed and used in this study.

File Name: Supplementary Data 2
Description: Expression vectors and transfection mixtures used in the Main Figures.

File Name: Supplementary Data 3
Description: Expression vectors and transfection mixtures used in Supplementary Data Figures.
